# *Xenopus*: An alternative model system for identifying muco-active agents

**DOI:** 10.1371/journal.pone.0193310

**Published:** 2018-02-22

**Authors:** Hyo Jung Sim, Sang-Hyun Kim, Kyung-Jae Myung, Taejoon Kwon, Hyun-Shik Lee, Tae Joo Park

**Affiliations:** 1 School of Life Sciences, Ulsan National Institute of Science and Technology (UNIST), Ulsan, South Korea; 2 CMRI, Department of Pharmacology, School of Medicine, Kyungpook National University, Daegu, Republic of Korea; 3 Center for Genomic Integrity, Institute for Basic Science, Ulsan, Republic of Korea; 4 College of Natural Sciences, Kyungpook National University, Daegu, South Korea; University of California, Merced, UNITED STATES

## Abstract

The airway epithelium in human plays a central role as the first line of defense against environmental contaminants. Most respiratory diseases such as chronic obstructive pulmonary disease (COPD), asthma, and respiratory infections, disturb normal muco-ciliary functions by stimulating the hypersecretion of mucus. Several muco-active agents have been used to treat hypersecretion symptoms in patients. Current muco-active reagents control mucus secretion by modulating either airway inflammation, cholinergic parasympathetic nerve activities or by reducing the viscosity by cleaving crosslinking in mucin and digesting DNAs in mucus. However, none of the current medication regulates mucus secretion by directly targeting airway goblet cells. The major hurdle for screening potential muco-active agents that directly affect the goblet cells, is the unavailability of *in vivo* model systems suitable for high-throughput screening. In this study, we developed a high-throughput *in vivo* model system for identifying muco-active reagents using *Xenopus laevis* embryos. We tested mucus secretion under various conditions and developed a screening strategy to identify potential muco-regulators. Using this novel screening technique, we identified narasin as a potential muco-regulator. Narasin treatment of developing *Xenopus* embryos significantly reduced mucus secretion. Furthermore, the human lung epithelial cell line, Calu-3, responded similarly to narasin treatment, validating our technique for discovering muco-active reagents.

## Introduction

The muco-ciliary epithelium of the airway tract is one of the most vulnerable tissues which are constantly challenged by external pathogens or contaminants. The airway epithelium consists of several cell types, such as the mucus secreting goblet cells, multi-ciliated cells, and basal cells [1]. The goblet cells synthesize and secrete mucus components such as mucin protein (Muc5AC or Muc5B in mammals), whereas the cilia of the motile ciliated cells expectorate the mucoid fluid, and the basal cells possibly possess a differentiation potential [1]. Mucus hypersecretion is one of the major symptoms of respiratory diseases such as asthma, chronic obstructive pulmonary disease (COPD), cystic fibrosis and respiratory infections [2]. Most medications for reducing mucus hypersecretion either alleviate inflammation, indirectly regulate mucus secretion as in the case of steroids, or reduce the viscosity of mucus to facilitate expectoration by reducing the disulfide bond of mucin as in the case of N-acetylcysteine or by digesting DNAs in the mucus as in the case of dornase [3]. Current experimental models for studying the activities of muco-regulators, including mouse tracheal primary cultures and human bronchial epithelial culture, are excellent for investigating the pathophysiology of mucus production and secretion. However, these techniques are labor, resource, and time-intensive [4]. Thus, current experimental models are not adequate for unbiased high throughput screening-based and unbiased drug discovery.

The *Xenopus* embryonic epidermis is an emerging *in vivo* model system with several advantages for studying the muco-ciliary epithelium. The embryonic epidermis of *Xenopus* is composed of multiciliated cells, goblet cells, ionocytes, and small secretory cells [5]. The similarity of the multiciliated cells of the *Xenopus* embryonic epidermis to those of mammalian multiciliated cells facilitated research on muticilia formation [6, 7]. Although the Muc genes in amphibians are highly evolved, differ from those of mammals, and are yet to be fully characterized [8], recent studies have further verified that the goblet cells of the *Xenopus* embryonic epidermis are tractable, and constitute an accessible model for investigating mucus secretion [5, 9, 10].

In this study, we developed a unique high-throughput screening strategy for identifying muco-active reagents using *Xenopus laevis* embryos. Using this unbiased screening technique, we showed that several known muco-regulators function in similar ways in the *Xenopus* muco-ciliary epithelium. Furthermore, we identified a new muco-active reagent, narasin. Narasin is a potent inhibitor of mucus secretion in both *Xenopus* epidermis and the human lung epithelial cell line, Calu-3. We believe that the screening technique developed in this study would benefit researchers studying the pathophysiology of mucus-related disorders.

## Materials and methods

### Ethics statement

The Animal Care and Use Committee from the Institutional Review Board of Ulsan National Institute of Science and Technology (UNIST) approved this work (Reference number, UNISTACUC-16-14). This study was performed in accordance with documented standards of the Animal Care and Use Committee from the Institutional Review Board of UNIST. All members of the research group attended educational and training courses for care and use of experimental animals. Adult *Xenopus laevis* were housed under 12-h light/dark cycles at 18°C in containers according to the guidance of Animal Care and Use Committee from the Institutional Review Board of UNIST.

### *Xenopus* embryo manipulation

Adult *Xenopus laevis* were obtained from Nasco (U.S.A). The average age of the *Xenopus laevis* is less than 5 years old and frogs were fed three times in a week with trout pellets from a local supplier. *Xenopus* were housed in the XR4 husbandry system (aquatic habitats) with a 12-hour light and 12-hour dark cycle. 12 frogs were housed in a 40 liter tank. The water quality for the system was set to the manufacturer’s recommendation (Temperature; 18°C, Conductivity; 1800 μS, pH; 7.0). Human chorionic gonadotropin (HCG) was used to trigger ovulation in an adult female one day before the experiments and the animal was squeezed to release the oocytes. A dissected testis from an adult male was used to fertilize the ovulated eggs. Cysteine solution (2.5%, pH 7.9) was used to remove the jelly layer encasing the eggs and the fertilized eggs were grown in 1/3× MMR until stage 32. For the ELLA (Enzyme-Linked Lectin Assay) analysis, four of the St. 32 embryos were nourished in 48 multi-well plates and the media from the wells were collected for further ELLA analysis. At least 5 independent experiments were performed for each condition.

### WGA-HRP-based mucus measurement

Stage 32 sibling embryos were washed vigorously several times in 1/3× MMR (33mM NaCl, 0.6mM KCl, 0.3mM MgCl_2_, 1.6mM HEPES) to clear the secreted mucus particles. After washing, the embryos were incubated for 3 hours in 1/3× MMR containing each library compound in a 48-well plate with a mesh-attached chamber. We used the SCREEN-WELL natural product library (Enzo BML-2865) and 10 mM Diversity Set provided by the National Cancer Institute’s (NCI) Developmental Therapeutic Program's Open Compound Repository. Then, the treated embryos were moved into fresh media without the compounds for an hour, following which the media was collected into a 96-well plate with a U-shaped bottom. The collected media was incubated at 4°C overnight. After incubation, the 96-well plate was washed twice with PTW (1× PBS, 0.1% Tween-20), followed by incubation in 1% bovine serum albumin (BSA) for an hour. After incubation, HRP-conjugated wheat germ agglutinin (WGA) (diluted as 1/10,000 in 1% BSA) was added and incubated for an hour. After binding, the samples were washed twice with PTW. Then, one tablet of o-phenylenediamine dihydrochloride (Sigma P8287) in phosphate citrate buffer (0.1 M dibasic sodium phosphate, 0.05 M sodium citrate, pH 5.0) was used for chromogenic detection. The reaction was stopped using 2.5 N sulfuric acid. The intensity was measured using a microplate reader at 492 nm. Z score was used to calculate the differences in detected mucus level with respect to the average, which was set at zero.

The Z-score is calculated as in the formula below:

Z-score = (x– μ) / σ

x; Raw measurement, μ; Mean, σ; Standard deviation

### Immunofluorescence and microscopy

For immunofluorescence microscopy, the embryos were fixed using MEMFA (1× minimum essential medium (MEM) salt, 4% formaldehyde) solution. The fixed embryos were washed with TBST (1× Tris-buffered saline, 0.1% Triton X-100) and then incubated in a blocking solution (10% fetal bovine serum (FBS), 2% dimethyl sulfoxide in TBST) for 30 minutes at room temperature, followed by incubation with primary antibodies at 4°C overnight. Next, the embryos were washed with TBST and incubated with secondary antibodies for an hour. Immunofluorescence analysis was performed with the following antibodies: anti-GFP (Abcam ab13970), anti-α-tubulin (DSHB 6G7), anti-actin (DSHB JLA20), WGA-Alexa 488 (Molecular probes, W11261), and anti-Muc5ac (Abcam ab77576) as primary antibodies. The stained embryos were imaged using a confocal microscope (Zeiss LSM880). Images were analyzed using Zen (Zeiss).

### Cell culture

Human lung epithelial cells, Calu-3 (ATCC HRB-55), were cultured in Dulbecco’s Modified Eagle’s medium (DMEM) (Lonza 12-604F). For RT-PCR, the cells were split into six-well plates and harvested for RNA extraction. For immunofluorescence analysis, the cells were split on a coverslip in six-well plates. Thapsigargin (1 μM) and narasin (2 μM and 4 μM) were used. After 3 hours of incubation, the cells were harvested for RT- PCR or fixed using 4% formaldehyde.

### Quantitative RT-PCR

Total RNA was extracted using a PureLink RNA mini kit (Invitrogen 12183018A), and the cDNA was synthesized using a random primer (NEB S1330S) and GoScript Reverse Transcriptase (Promega A50047). Quantitative RT-PCR was performed using the LightCycler 480 instrument (Roche LC480). The expression of ER stress marker genes was used to assess ER stress levels after normalization to *GAPDH* expression (internal control) (GAPDH forward: 5′-GGCCTCCAAGGAGTAAGACC-3′, backward: 5′-AGGGGTCTACATGGCAACTG-3′; CHOP forward: 5′-GGAGCATCAGTCCCCCACTT-3′, backward: 5′-TGTGGGATTGAGGGTCACATC-3′; IRE1 forward: 5′-TGCTTAAGGACATGGCTACCATCA-3′, backward: 5′-CTGGAACTGCTGGTGCTGGA-3′; PERK forward: 5′-AATGCCTGGGACGTGGTGGC-3′, backward: 5′-TGGTGGTGCTTCGAGCCAGG-3′; spliced XBP1 forward: 5′-CGCTTGGGGATGGATGCCCTG-3′, backward: 5′-CCTGCACCTGCTGCGGACT-3′; total XBP1 forward: 5′-GGCATCCTGGCTTGCCTCCA-3′, backward: 5′-GCCCCCTCAGCAGGTGTTCC-3′).

## Results

### 1. *Xenopus laevis*: An alternative model system for studying muco-ciliary epithelium

**Fig 1:**
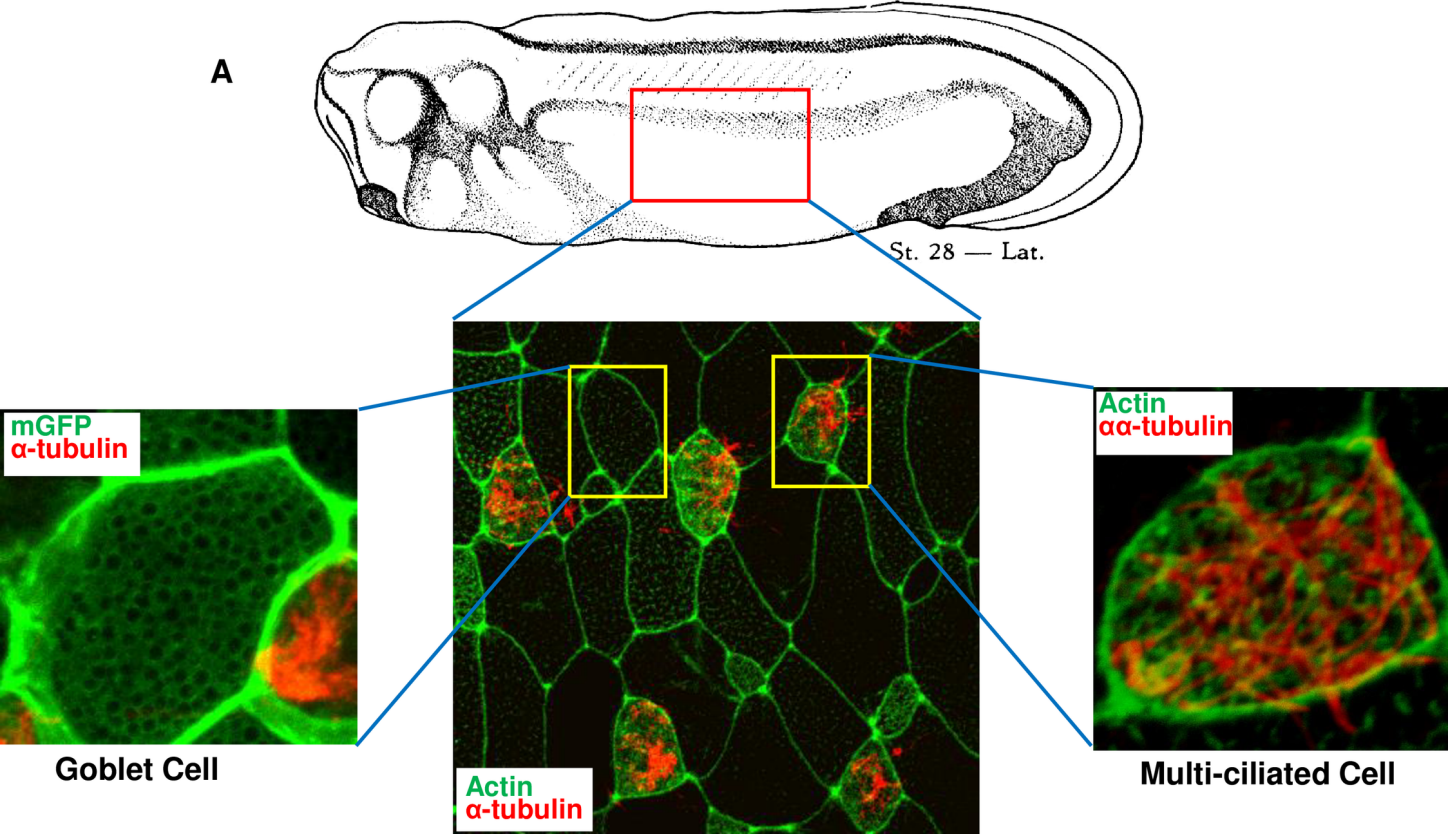
The composition of the muco-ciliary epithelium of *Xenopus laevis* embryo. **A.** Immunofluorescence image of mucus secreting goblet cells and multiciliated cells in *Xenopus* embryonic epidermis. The membrane was stained using membrane GFP (green), Cilia were detected using α-tubulin (red) and actin by anti-actin antibody (green).

The mouse tracheal epithelium and human bronchial cells are frequently used to study the muco-ciliary epithelium. The mouse model is well-established as a means of exploring the pathophysiology of the muco-ciliary epithelium; however, *in vivo* mouse models or primary air-liquid interface (ALI) culture systems require cautious handling and a considerable amount of resources. Cancer cell lines provide alternative and compensatory models for studying the muco-ciliary epithelium; however, cancer cell lines are not physiologically relevant in certain contexts. Therefore, the muco-ciliary epithelium of *Xenopus* embryonic tissue is an excellent alternative model system that provides an accessible source of tractable tissues for investigating the pathophysiology of human respiratory disorders [9, 11]. The goblet cells constitute approximately 50% of the *Xenopus* embryonic epidermal cells (Fig 1). The genetic and physiological characteristics of *Xenopus* goblet cells are very similar to those of human tracheal goblet cells [9, 12]. *Xenopus* goblet cells secrete mucins and ions to protect the embryonic skin from environmental contaminants [5]. The secreted mucus components are constantly washed off in the surrounding media. In addition, *Xenopus* embryos do not require a complex serum-based culture media and can be nourished in minimally salted water, which is suitable for the detection of mucus components in the media using enzyme-linked lectin assay (ELLA)-based methods.

### 2. *In vivo* screening system using *Xenopus laevis*

**Fig 2:**
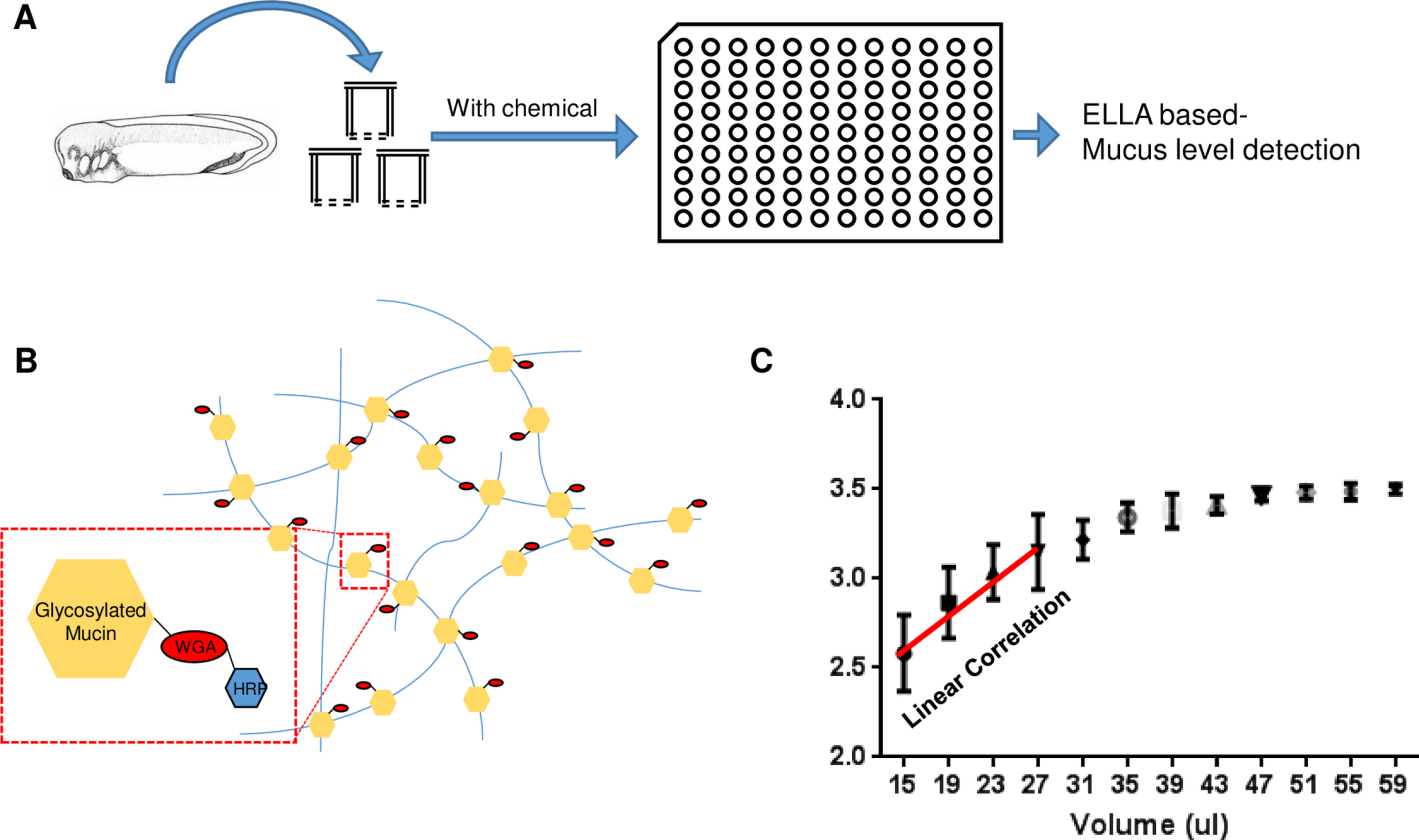
High throughput screening strategy for muco-active reagents from the compounds library using *Xenopus* embryos. **A.** Schematic showing the drug screening procedure. Sibling *Xenopus* embryos were incubated with individual compounds in the library. Then, the media was collected after washing off the compound from the media. The mucus in the media was detected using WGA-HRP and mucus level was analyzed by measuring the optical density (OD) of the HRP substrates. **B.** Schematic showing the mucus detection procedure using WGA-HRP in ELLA. WGA-HRP can detect the glycosylated region of mucus in the media. **C.** Dose-dependent linear correlation of OD values with the amount of media used for mucus detection in ELLA.

Owing to the complexity of culture conditions and detection protocols, current research models such as primary cells cultured at the air-liquid interface or immortalized cell lines are not suitable for unbiased massive screening for muco-active regents [4]. The abundance and simple culture conditions of the embryonic muco-ciliary epidermis of *Xenopus* embryos may provide a suitable alternative for high-throughput screening-based strategies. For identification of muco-active reagents from unbiased chemical libraries using a high throughput approach, we first examined if the secreted mucus level in *Xenopus* embryo culture media can be efficiently detected by ELLA (Fig 2A). The major components of secreted mucus are glycosylated proteins such as mucins. Lectins specifically bind to glycosylated oligosaccharides and have been frequently used to detect glycosylated proteins in mucus [4]. In this method, the secreted mucus in the media is detected directly using horseradish peroxidase-fused wheat germ agglutinin (WGA-HRP), a lectin. WGA specifically binds to the N-acetylglucosamine residues on glycosylated proteins and has been used to determine mucus levels in human airway epithelium [4]. HRP was used for colorimetric reaction and detection (Fig 2B).

We cultured *Xenopus* embryos in a multi-well plate and collected the media to determine levels of secreted glycoprotein. Since the *Xenopus* culture media contains minimal salt and no serum-based complex medium and proteins, ELLA was more suitable for the accurate detection of secreted glycoproteins. Indeed, the ELLA absorbance values show a linear correlation with increasing doses of culture media in a certain range (Fig 2C).

### 3. The *in vivo* screening system yielded several muco-active reagents

**Fig 3:**
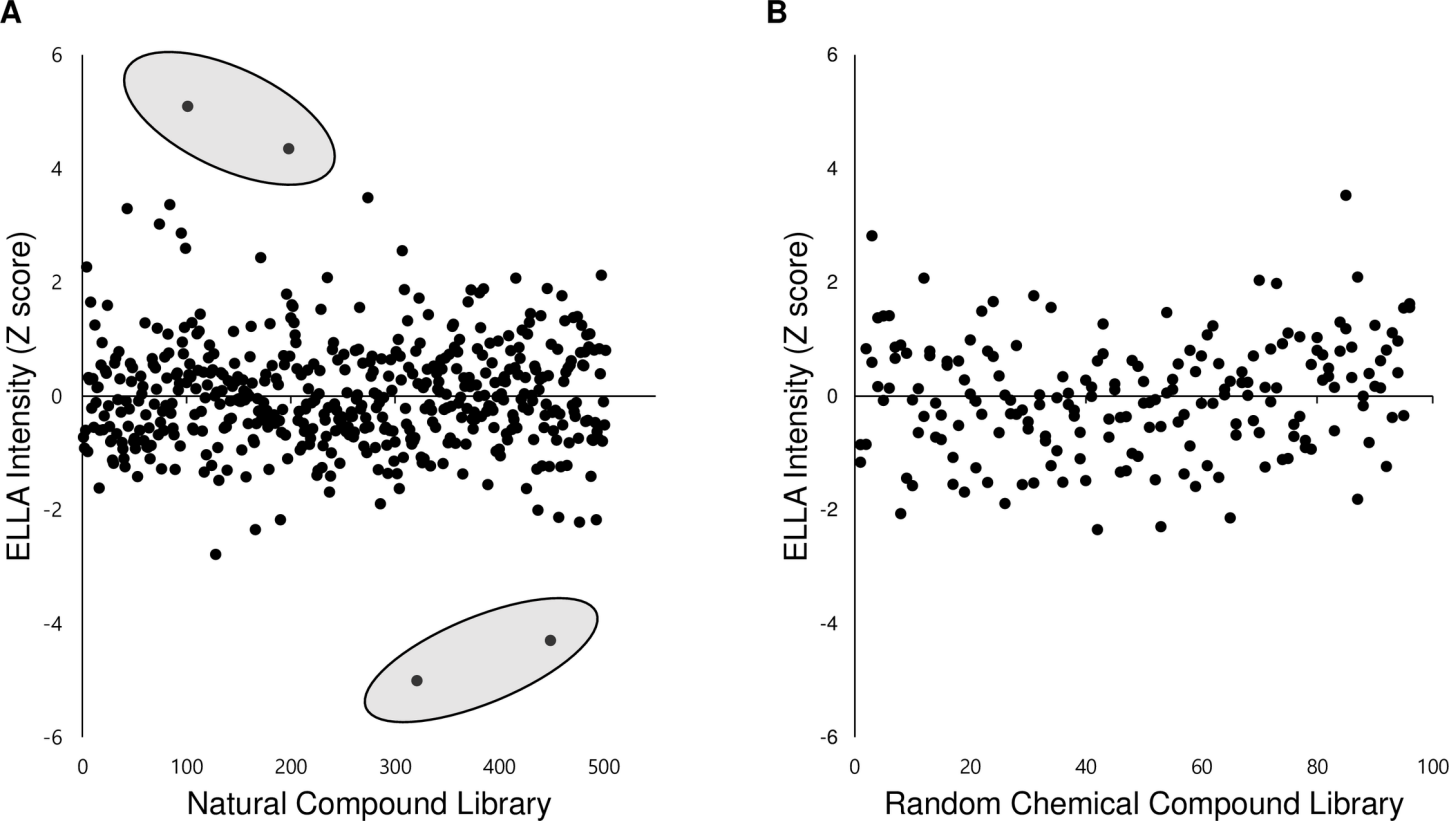
High throughput screening yielded putative muco-active reagents from the compounds library. **A.** The result of natural compound library screening. Data were analyzed by calculating the Z-score which indicates how far an individual score is away from the mean of a data set. Four putative muco-active reagents which have a Z-score over 4 or below -4 were selected for further analysis. **B.** The screening results of a random chemical library from NCBI. The random chemical library did not yield any muco-active reagents.

Since the *in vivo* screening system using *Xenopus* embryos can be used to efficiently analyze the mucus secretion level, we examined a SCREEN-WELL natural product library (Enzo BML-2865) and a 10 mM Diversity Set provided by the National Cancer Institute’s (NCI) Developmental Therapeutic Program's Open Compound Repository, to identify compounds that regulate mucus secretion. The final concentration of the chemicals used was 15 μM. Embryos were incubated in the media with individual chemicals for 3 hours, after which they were moved to new media which were without the chemicals. After 1 hour of further incubation, the media was collected for ELLA based-mucus detection. The graph represents the ratio of optical density measurements of drug-treated media and the averaged control media in ELLA. Data were analyzed by calculating the Z-score representing the differences of individual values from the mean value. Using this new strategy, we tested approximately 600 natural compounds and 200 synthetic chemicals. While none of the chemical compounds from the Diversity Set provided by the National Cancer Institute made any significant changes in ELLA signal intensity, the natural compound library yielded several compounds that potentially affected mucus levels (Fig 3A and 3B).

### 4. Identification of possible muco-regulators

**Fig 4:**
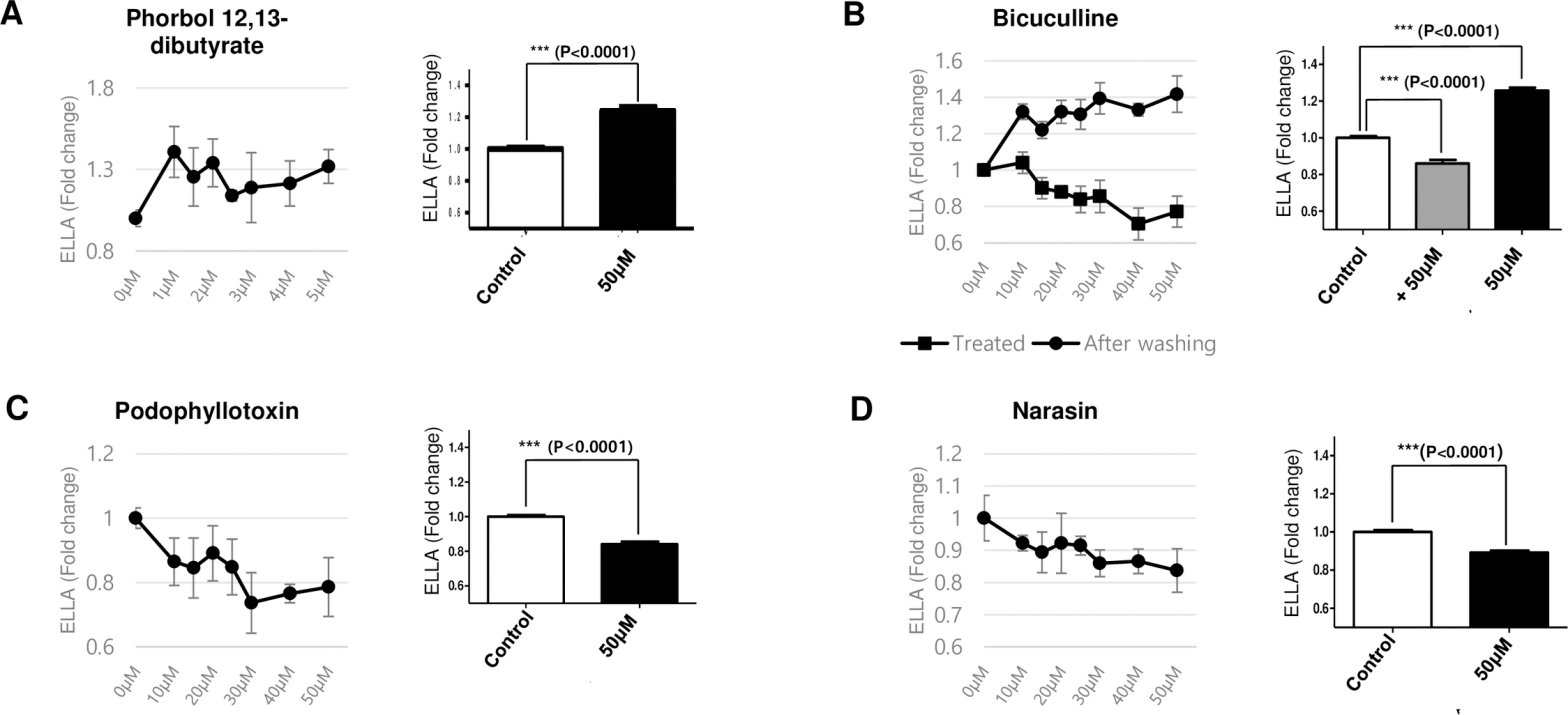
Putative muco-active reagents affecting mucus secretion level. The mucus level in the media upon the treatment of increasing doses of individual chemicals was normalized to those of control embryos and plotted on the graph (left panel, n = 5). The secreted mucus levels at 50uM of each natural compound were plotted (right panel, n = 39). P-values were calculated from student’s t-test. **A.** Phorbol 12,13-dibutyrate is a known activator of PKC and promotes mucus secretion. **B.** Bicuculline is a GABA antagonist and inhibits mucus secretion. In the presence of bicuculline, mucus secretion was strongly inhibited (rectangle line); however, mucus secretion recovered after removing bicuculline (circle line). **C.** Podophyllotoxin is a known microtubule polymerization inhibitor with anti-viral and anti-tumor activities. Podophyllotoxin strongly inhibited mucus secretion from the *Xenopus* embryonic epithelium. **D.** An ionophoric coccidiostat, narasin inhibits mucus secretion.

Next, we selected four natural compounds that significantly affected the mucus secretion level and systemically examined the muco-regulating activities of the individual compounds. To investigate the effect of the selected candidates on mucus secretion in further detail, the cells were treated with various doses of the chemicals and the secreted mucus levels were determined using the WGA-HRP-based ELLA protocol. Indeed, all four candidates considerably affected mucus secretion from the *Xenopus* embryonic epidermis (Fig 4).

Phorbol 12, 13-dibutyrate and bicuculline increased, whereas phodophyllotoxin and narasin decreased the secreted mucus level in the media (Fig 4). Among these chemicals, phorbol 12, 13-dibutyrate was previously reported to increase glycoprotein secretion from goblet cells by activating protein kinase C (PKC) [13, 14]. Bicuculline was also previously reported to regulate mucus secretion. Interestingly, however, bicuculline is known to inhibit mucus secretion by antagonizing GABA signaling [15]. We speculated that bicuculline may reversibly inhibit mucus secretion, and the retained mucus is possibly released in the medium after washing off bicuculline. Indeed, the secreted mucus level was reduced in the presence of bicuculline; however, mucus secretion increased after exchanging the media with bicuculline-free media (Fig 4B).

**Fig 5:**
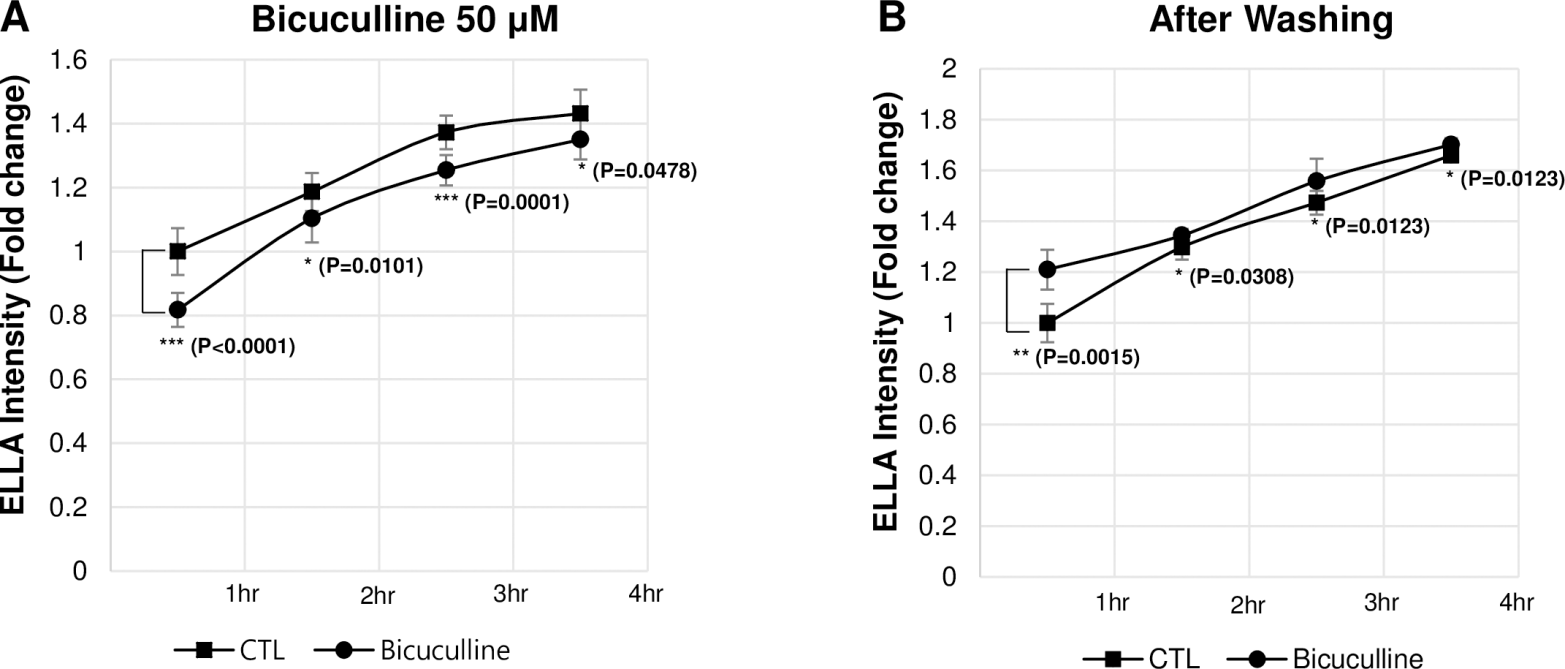
Bicuculline reversibly inhibits mucus secretion. Mucus secretion was analyzed in the presence of 50uM of bicuculline **(A)** or after changing the media without bicuculline at every 30 min **(B)**. Mucus secretion was restored within 1 hour after removing bicuculline. P-values were calculated from student’s t-test (n = 4).

Also, we performed a time-course analysis of the mucus secretion upon the treatment of the bicuculline and after removing bicuculline respectively (Fig 5). Indeed, the reduced mucus secretion by treating bicuculline, was recovered within one hour after removing bicuculline. These results show that the physiological properties of the *Xenopus* muco-ciliary epidermis are similar to those of the mammalian airway epithelium, and that our screening protocol can effectively and unbiasedly detect muco-active reagents.

### 5. Narasin disrupts mucus secretion and causes mucus accumulation in the intracellular compartment

**Fig 6:**
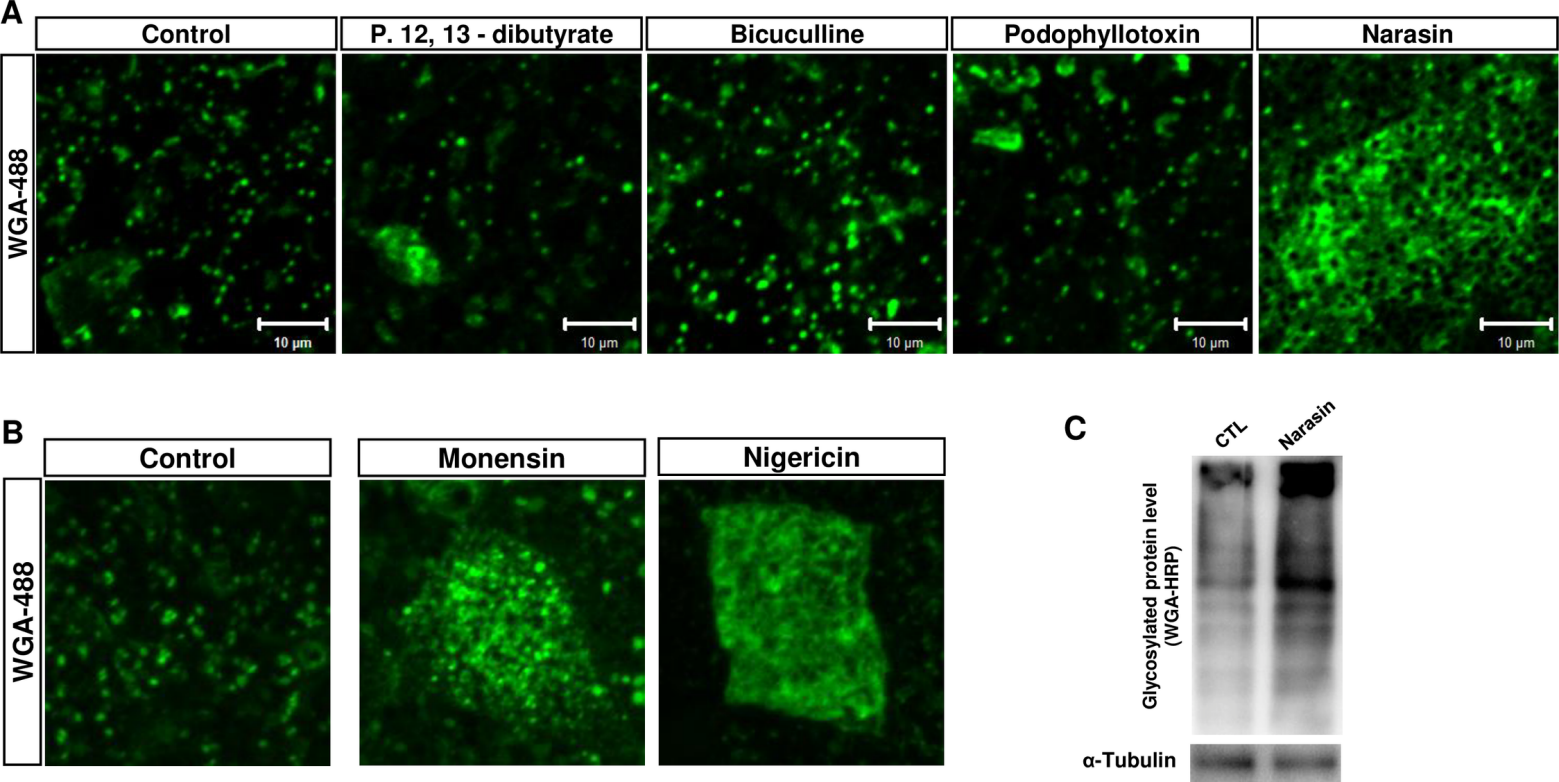
Ionophoric antibiotics narasin inhibits mucus secretion. **A.** The mucus granules were stained with WGA-Alexa 488 to visualize retained mucus granules. Narasin treated goblet cells (last panel) displayed severe retention of mucus granules. **B.** Ionophoric antibiotics, Monensin and Nigericin, inhibit mucus secretion similar to narasin. **C.** Narasin treatment increased intracellular levels of glycosylated protein.

We further analyzed the changes in mucus granules in goblet cells after treating these four muco-active chemicals to the *Xenopus* embryos by staining them with WGA-Alexa 488 (Fig 6A). Among the four muco-active chemicals, the retention of mucus in narasin-treated goblet cells was particularly interesting (Fig 6A, left panel). Narasin is an ionophoric coccidiostat used to treat and protect poultry animals from coccidial infections. We further examined mucus retention after treating other known coccidiostats such as monensin and nigericin. Indeed, both monensin and nigericin accumulated mucus granules in the goblet cells of *Xenopus* epidermis (Fig 6B). We confirmed the mucus retention by performing western blot analysis using WGA-HRP (Fig 6C).

**Fig 7:**
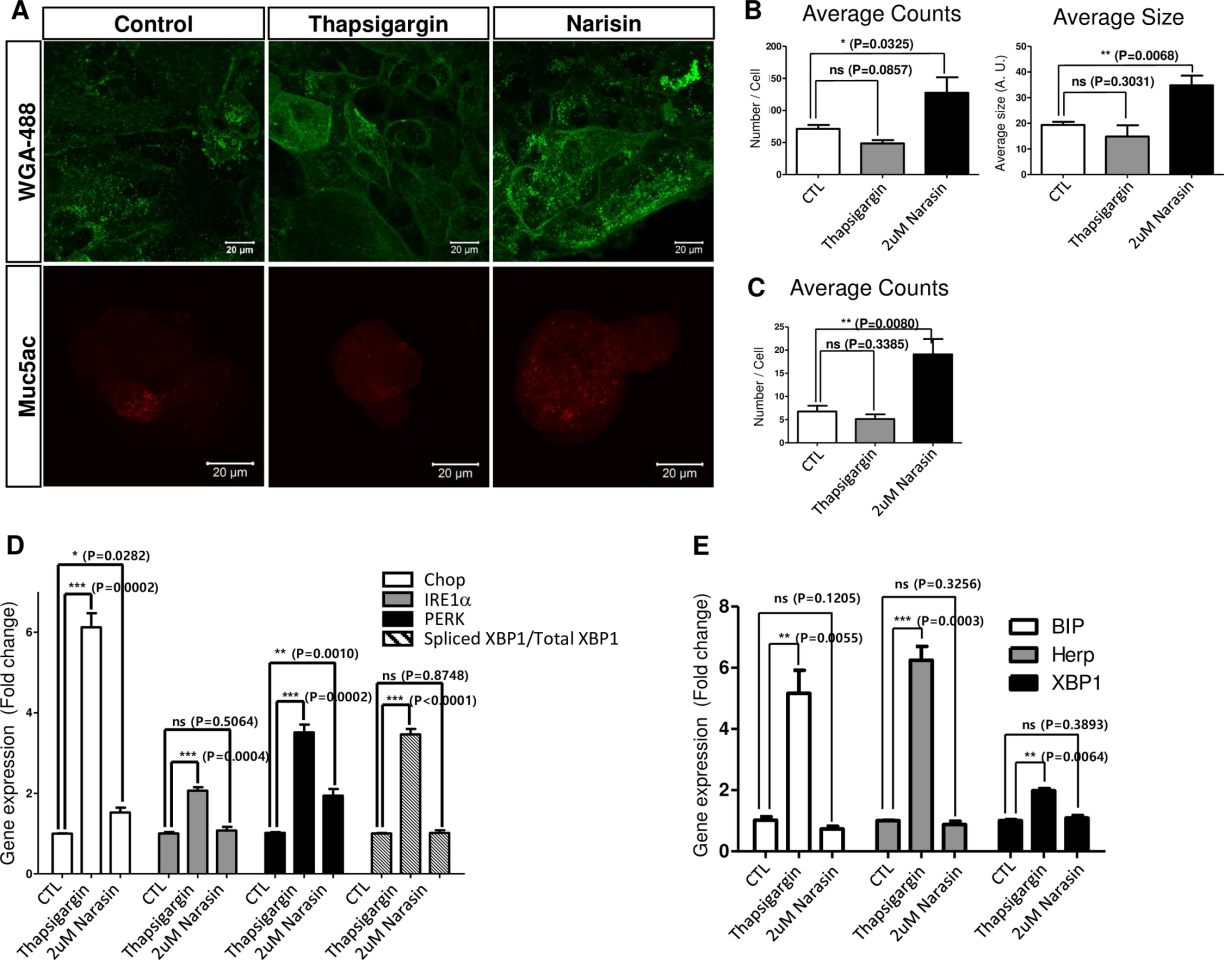
Narasin inhibits mucus secretion in Calu-3 cells. **A.** Narasin-treated Calu-3 cells were stained with WGA (green) and Muc5ac (Red). Narasin-treated Calu-3 cells accumulated Muc5ac-positive puncta and WGA-stained glycoproteins in the cytosol. **B.** The number and average size of WGA stained signal was plotted graphically. **C.** The number of Muc5ac stained puncta was measured and represented graphically. **D.** Although the PERK level was slightly increased, ER stress was not strongly induced by narasin in Calu-3 cells. Thapsigargin induced strong ER stress response but mucus secretion was not affected. **E.** Narasin did not severely induce ER stress responses in *Xenopus* embryos. P-values were calculated from student’s t-test using the triplicate Q-PCR data set. A.U.; Arbitrary unit.

The muco-active functions of narasin were not previously reported. Therefore, we further assessed narasin’s muco-regulatory activity using human mucus-secreting Calu-3 cells. The Calu-3 cells are frequently used to study mucus secretion *in vitro* [16]. We treated Calu-3 cells with narasin for 2 hours and determined the intracellular glycoprotein levels by analyzing the WGA-Alexa488 fluorescent signals. Indeed, the intensity of the WGA-Alexa488 signal was significantly higher in narasin-treated Calu-3 cells than in the control cells (Fig 7A and 7B). Next, we used immunofluorescence imaging analysis to determine whether Muc5ac also accumulated intracellularly in narasin-treated cells. As expected, the narasin-treated Calu-3 cells accumulated larger quantities of Muc5ac than the mock-treated Calu-3 cells (Fig 7A and 7C).

Finally, we sought to understand the underlying mechanism of narasin’s role in mucus retention. Narasin exposure is known to increase endoplasmic reticulum (ER) stress in cancer cells [17]. We speculated that an increase in ER stress may perturb normal secretory pathways or Muc5ac processing upon narasin treatment. We monitored changes in ER stress signals after narasin treatment. However, as shown by comparison to the control samples, narasin treatment did not strongly affect ER stress levels in either Calu-3 cells (Fig 7D) or *Xenopus* (Fig 7E). In addition, thapsigargin treatment, which is known to increase ER stress responses, did not strongly alter intracellular mucus levels of Muc5AC and glycoproteins (Fig 7A, 7B and 7C), although it significantly increased ER stress signals (Fig 7D and 7E), which were assessed by measuring the expression of ER stress response genes using quantitative real time-polymerase chain reaction (RT-PCR). These results suggest that narasin may directly affect mucus secretion, which excludes the involvement of ER stress-associated secondary mucus production.

## Discussion

In this study, we exploited the advantages of the *Xenopus* embryonic epithelium to develop an unbiased drug discovery strategy for novel muco-active reagents. Indeed, our novel technique efficiently and effectively detected changes in mucus levels in the nourishing media by a simple two-step analysis procedure involving WGA-HRP detection and chromogenic measurement. Furthermore, we tested the muco-regulatory activities of a natural compound library to validate our high throughput screening procedure, which is currently challenging in other popular model systems such as the HBEC ALI culture or mouse models. We have identified several muco-active reagents from this pioneering natural compound library screening. Among several candidates, two chemicals, phorbol 12,13-dibutyrate and bicuculline, are known to control mucus secretion as a PKC activator and GABA receptor antagonist respectively. These data validated the effectiveness of our technique. In addition, we identified a novel muco-active compound called narasin, which can possibly be used as a therapeutic for respiratory diseases. Genomic analysis in *Xenopus tropicalis*, revealed a huge expansion of Muc genes in its genome [8]. Apparently, *Xenopus* genome contains 26 gel forming mucin genes including 12 Muc2 genes (Muc2A-L), and 11 Muc5 genes (Muc5A-K). Although, the Muc genes in *Xenopus* are highly evolved and differ structurally from those of humans, our data suggest the secretory mechanism may be conserved in *Xenopus* and can be used to discover the muco-regulator affecting mucus secretion from the goblet cells. Further research on the physiology and molecular mechanism of Muc gene regulation in *Xenopus* would strengthen the utility of embryonic epidermis as an alternative model system to study mucociliary epithelium.

Abnormalities in muco-regulation are one of the most frequently observed symptoms in respiratory disorders. Therefore, novel reagents that effectively regulate mucus secretion from the airway epithelium are being actively researched. However, current muco-regulation research models do not provide high throughput screening protocols for identification of muco-regulators in an unbiased manner. In this study, we developed a cost-efficient and unbiased strategy targeting muco-active reagents using *Xenopus* embryonic epidermis.

The embryonic epidermis of *Xenopus* has been extensively used to study multicilia as a tractable and alternative model. In addition, several types of secretory cells such as the goblet cells, ionocytes, and small secretory cells are also in the limelight for research on muco-regulation. The *Xenopus* embryonic epidermis possesses several advantages compared to other research models such as human bronchial epithelial cells (HBEC) and the mouse model. One *Xenopus* female can produce thousands of embryos per day, which generates an unlimited source of research material. Unlike HEBCs, which take several weeks to differentiate into the muco-ciliary epithelium in ALI culture, the *Xenopus* embryonic epidermis differentiates to a completely functional muco-ciliary epithelium within two days. The mouse airway epithelium is an excellent *in vivo* model; however, internal organs are not suitable for massive drug screening purposes, as they require a substantial amount of labor and resources. In contrast, the *Xenopus* embryonic epidermis is located on the external skin of embryos, and is favorable for experimentally analyzing muco-regulation. Moreover, *Xenopus* embryos grow in minimally salted water and the secreted mucus can be accurately and simply measured using ELLA.

Narasin is an ionophoric antibiotic used for treating coccidial infections in poultry and livestock. Although narasin is widely used in farm animal feed, its cytotoxic effects on several organs such as the cardiovascular, muscular, and gastrointestinal systems have been reported. However, the underlying mechanism of its cytotoxicity is not yet understood. In this study, we observed that narasin severely alters mucus secretion and induces accumulation of mucus granules in the cytosol. Our data suggest that the inhibitory effect of narasin on mucus secretion is not due to secondary effects stemming from ER stress responses, which was previously shown to be induced by narasin in a human cell line. Extensive research is required to understand the underlying mechanisms of the muco-regulatory activity of narasin.

The muco-ciliary epithelium of the airway tract is one of the most vulnerable tissues of the human body and mucus hypersecretion is one of the major symptoms of respiratory disorders. We believe that the high throughput screening strategy developed in this study will accelerate the identification of effective muco-regulators with novel functions and mechanisms.
